# Osteogenic potential of induced pluripotent stem cells from human adipose-derived stem cells

**DOI:** 10.1186/s13287-019-1402-y

**Published:** 2019-10-17

**Authors:** Shih-Hsuan Mao, Chih-Hao Chen, Chien-Tzung Chen

**Affiliations:** 1 Craniofacial Research Center and Department of Plastic and Reconstructive Surgery, Chang Gung Memorial Hospital at Linkou, Chang Gung University, College of Medicine, Taoyuan, 333 Taiwan; 2 Department of Plastic and Reconstructive Surgery, Chang Gung Memorial Hospital at Keelung, Chang Gung University, College of Medicine, Keelung, 204 Taiwan

**Keywords:** Induced pluripotent stem cells, Adipose-derived stem cells, Bone tissue engineering, Cryogel, Osteogenic differentiation

## Abstract

**Background:**

Bone regeneration is a crucial and challenging issue in clinical practice. Bone tissue engineering (BTE) with an optimal cell source may provide an ideal strategy for the reconstruction of bone defects. This study examined whether induced pluripotent stem cells (iPSCs) derived from adipose-derived stem cells (ASCs) could act as an osteogenic substitute and whether these ASC-iPSCs yield more new bone formation than ASCs in hydrogel scaffolds.

**Methods:**

ASC-iPSCs were reprogrammed from ASCs through a retroviral system. ASCs were harvested and isolated from adipose tissue of humans. An aliquot of cell suspension (1 × 10^6^ cells/mL) was seeded directly onto the nHAP-gelatin cryogel scaffolds. Nude mice back implantation of cell-seeded scaffolds was designed for in vivo comparison of osteogenic potentials between ASCs and ASC-iPSCs. Samples were harvested 4 and 8 weeks after implantation for further analysis based on histology and RT-PCR.

**Results:**

ASC-iPSCs were successfully obtained from human adipose-derived stem cells. PCR results also showed that specific genes of iPSCs with the ability to cause the differentiation of cells into the three germ layers were expressed. In our in vivo experiments, iPSCs were subcutaneously injected into nude mice to induce teratoma formation. The morphology of the three germ layers was confirmed by histological staining. ASC is an essential cell source for BTE with benefits of high volume and less-invasive acquisition. With additional transforming Yamanaka factors, ASC-iPSCs showed higher osteogenic differentiation and elevated expression of collagen type I (Col I), osteocalcin (OCN), alkaline phosphate (ALP), and runt-related transcription factor 2 (RunX-2).

**Conclusions:**

This report suggests that ASC-iPSCs could be a superior cell source in BTE with better osteogenic differentiation efficacy for future clinical applications.

## Background

Bone regeneration is a crucial issue in clinical practice. Various etiologies, such as degeneration, trauma, congenital anomalies, or oncological resection, would result in bony destruction and subsequent defects causing significant morbidities and functional limitations. These defects can occur in different regions like craniofacial area or extremities. With decades of progression in reconstructive surgery, vascularized or non-vascularized autologous tissue transfer provides possible therapeutic approaches [1, 2]. However, the reconstructive modalities are restricted because of potential donor site morbidity and limited sources for larger defects. Bone tissue engineering (BTE) sheds a light on the unmet need. As current understandings, a successful model of tissue engineering requires adequate cell sources, scaffold, and growth factors. The ideal cell source should have advantages of minimal morbidity, rapid expansion, and designated differentiation along with adequate quantity and function. Previous studies demonstrated the feasibility of BTE as a potential alternative to reconstruct bone defect, especially in craniofacial regions [3]. Nevertheless, lack of sufficient and constant cell sources preclude the expansion of BTE in clinical practices.

Several sources of mesenchymal stem cells derived from bone marrow, adipose, or umbilical cord have been shown as potential sources for BTE. Bone marrow mesenchymal stem cells (BM-MSCs) share a close lineage to osteogenesis. However, the acquisition procedure by bone marrow aspiration is invasive, and the cell quantities could be insufficient. Adipose-derived stem cells (ASCs) are pluripotent MSCs with multilineage differentiation of ectoderm, mesoderm, and endoderm [4–7]. ASCs possess advantages including harvest in a large quantity under a less invasive procedure. Despite the versatility of differentiation, the efficacy of osteogenic differentiation in ASC is less favorable compared with BM-MSCs [8]. Takahashi and Yamanaka introduced the induced pluripotent stem cells (iPSCs) by transforming embryonic fibroblasts into pluripotency embryonic cells toward disease and patient-specific cell sources [9]. The breakthrough provides an alternative cell source in tissue engineering. However, the procedure is lengthy in expansion, has low reprogramming efficiency, and has risk of contamination by feeder layers which limit the application clinically. Sun et al. and Sugii et al. demonstrated a potential solution by utilizing transforming ASCs into ASC-iPSCs with and without feeder layers [10, 11]. This innovation might further improve the efficacy and accessibility in clinical settings. Preclinical studies were conducted by transforming adipose stromal cells into iPSCs and derived endothelial cells to treat myocardial infarctions and limb ischemia [12, 13].

In this study, we would like to refine the efficacy of osteogenic differentiation in ASCs by transforming Yamanaka factors. We examine and compare the ability of ASCs and ASC-iPSCs in proliferation and osteogenic differentiation.

## Materials and methods

### Isolation of human adipose-derived stem cells (ASCs)

The study was approved by the institutional Review Board of Chang Gung Memorial Hospital. Patients’ consents were acquired before procedures. The fat was harvested by direct excision. The harvested fat was placed in a sterile container and transported to the laboratory on ice. The fat was cut into small lobules and homogenously mixed with 0.075% collagenase type 1A at a ratio of 1:1 in volume for 30 min at 37 °C. The digestion was terminated by α-MEM in equal volume. The solution was centrifuged with 2000 rpm for 5 min under 37 °C. The upper solution was discarded. Another 10 mL of α-MEM medium was mixed with a cell solution. The cell solution was filtered by 70-μm cell strainers followed by repeating centrifuge with 2000 rpm for 5 min under 37 °C. The upper solution was discarded. The centrifuged cell solution was incubated in 10-cm dishes under 37 °C and 5% CO_2_ for 16 h. The medium was replaced two to three times per week. Flow cytometry (Beckman Coulter Cyan ADPs) was utilized to preliminarily investigate expression of surface markers of ASC, including CD13, CD14, CD29, CD34, CD45, CD73, CD90, and CD105 according to the definition of mesenchymal stem cells according to the International Society for Cellular Therapy (ISCT). Multilineage differentiation potential of human ASCs was induced to differentiate into adipocytes, chondrocytes, and osteocytes using a previously described differentiation protocol. Oil Red O was used to stain adipocytes, Alcian blue was used to stain chondrocytes, and Alizarin red was used to stain osteocytes.

### Preparation of ASC-iPSCs

#### In vitro cell culture

ASC-iPSCs were reprogrammed according to protocols described previously [14]. Briefly, passages 1 to 3 of ASCs were reprogrammed into ASC-iPSCs via a retroviral system encoding four factor combinations, including OCT4 (POU class 5 homeobox 1), SOX2 (SRY-box 2), KLF4 (Kruppel-like factor 4), and c-MYC (myelocytomatosis oncogene). The reprogrammed human ASC-iPSCs were maintained on mitomycin C-treated mouse embryonic fibroblast (MEF) feeder cells in Knockout Dulbecco’s modified Eagle’s medium (DMEM, Invitrogen) supplemented with 20% knockout serum replacement (KSR; Invitrogen), 8 ng/mL bFGF (Propetech), 50 U/mL penicillin, 50 mg/mL streptomycin, 1 mM sodium pyruvate, 0.1 mM MEM non-essential amino acid, 2 mM l-glutamine, and 0.1 mM 2-mercaptoethanol, and passaged with 0.1% EDTA (Invitrogen) in PBS at a ratio of 1:3 every week. The efficacy of transfection of ASCs to ASC-iPSCs, OCT4, SOX2, c-MYC, KLF4, Nanog, and GAPDH was examined by RT-PCR. Marker protein of embryonic stem cells, including SSEA-4, Tra-81, and Nanog, was examined by immunofluorescence stains.

#### Teratoma formation

The animal protocols were approved by The Institutional Animal Care and Use Committee of Chang Gung University and in accordance with the standards of the Association for Assessment and Accreditation of Laboratory Animal Care. Ketamine (20 mg/kg) was administered through intramuscular injection for anesthesia before animal surgery. All procedures were carried out under sterilized equipment and sterilized hood. To examine the potential of multilineage differentiation of ASC-iPSCs, teratoma formation was conducted according to a previous report by Zhang et al. [15] Briefly, 1 × 10^6^ cells suspended with 1 mL of Martigel were injected subcutaneously into the hind limbs of 4-week-old male nude mice. After 14 weeks of observation, the mice were sacrificed with retrieval of teratomas which were examined by hematoxylin and eosin (H&E) stain.

### nHAP-gelatin cryogel preparation

The characteristics and preparation was previously described [16]. Briefly, pre-weighed nanohydroxyapatite (nHAP) nanoparticles were dispersed in a gelatin solution (8%) in an MES buffer (pH = 6.5) at 70 °C into a gelatin-nHAP suspension with 2% nHAP (solution A). EDC was dissolved in a 10-mL MES buffer (pH = 6.5) to reach an initial concentration of 0.02 M (solution B). Solutions A and B were mixed at an equal volume ratio to get a 4% gelatin/1% nHAP suspension in a bottom-capped 3-mL syringe (8.5 mm inner diameter) that served as the mold, and further mixed using a home-made vibration-free overhead spindle stirrer. The syringe mold was immersed in a 95% ethanol bath kept at − 16 °C for 16 h to complete the cryogelation process. The syringe mold was taken out of the bath after completion of the reaction, and the cryogel scaffold was allowed to recede through the bottom cap. The cryogel was cut into cylindrically shaped discs of 1-mm thickness, from which gel disks of 2 mm diameter × 1 mm thickness were made using a tissue puncher. All cryogel scaffolds were transferred to a − 80 °C freezer for 24 h and further lyophilized to obtain macroporous gelatin-nHAP cryogel scaffolds containing 20% (w/w) nHAP.

### In vivo study

#### Preparation of cells and scaffold

An aliquot of cell suspension (1 × 10^6^ cells/mL) was seeded directly onto the top surface of the wet cryogel disk. The cell-seeded cryogel was incubated at 37 °C in a CO_2_ incubator for 4 h to allow cell attachment and turned upside down for cell seeding to the other surface as before. After incubating for 4 h in a CO_2_ incubator, the cell-seeded scaffold was transferred to a new well in a 24-well cell culture plate and 2 mL of α-MEM containing 10% FBS and 1.0% streptomycin–penicillin solution was added to each well and cultured at 37 °C in humidified 5% CO_2_ for 3 days with medium change three times per week to allow complete attachment. Osteo-induction medium (10 mM β-gap, 10% FBS, 1% PSA, 50 mM vitamin C, 50 μM Dex in DMEM/F12 medium) was then added for the next 14 days with medium change three times per week.

#### Animal experiment

The animal protocols were approved by The Institutional Animal Care and Use Committee of Chang Gung University and in accordance with the standards of the Association for Assessment and Accreditation of Laboratory Animal Care. Ketamine (20 mg/kg) was administered through intramuscular injection for anesthesia before animal surgery. All procedures were carried out under sterilized equipment and sterilized hood.

The back skin of nude mice was carefully excised followed by implantation of cell-seeded scaffolds. Wound was closed primarily and dressed with antibiotic ointment to avoid infection. At 4 and 8 weeks, two animals from each group were euthanized with lethal doses of pentobarbital (0.5 g/kg body weight). The back skin was excised followed with implants dissected out for examination and photography. After gross evaluation, the samples were fixed in 10% formaldehyde, dehydrated in alcohol, and embedded in paraffin, and sections were examined histologically under ALP staining.

#### RT-PCR analysis

Total RNA of each specimen was isolated with GeneJET RNA Purification kit (Thermo) according to the manufacturer’s instructions. The primers for osteogenic differentiation were listed in Table 1. Isolated RNA was dissolved in RNase-free water, and the amount of RNA was determined by measuring absorbance at 260 nm with a spectrophotometer. Further, RNA quality was verified by agarose electrophoresis and the measurement of OD260/OD280. After that, the RNA samples were treated with DNase I (Thermo). The cDNA was prepared from 2 mg of total RNA with RevertAid First Strand cDNA Synthesis Kit (Thermo) in a final volume of 20 mL. For a single PCR reaction amounting to 20 mL, 0.1 mL of cDNA was used. The SYBR Green I supermix (Bio-Rad) was used for visualization of PCR products in real time. A two-temperature cycling, consisting of a denaturation step at 95 °C for 10 s and annealing/extension step at 52–68 °C for 30 s, was carried out in an iCycle instrument (Bio-Rad). The specificity of each PCR reaction was assessed by performing melting curve analysis after each reaction. To normalize for input load of cDNA between the samples, glyceraldehyde-3-phosphate dehydrogenase (GAPDH) was used as an endogenous standard. Each of the cDNA was tested in duplicate. The standard curve method was used to quantify the relative change fold between samples. The correlation coefficient of each standard curve was around 0.99.

**Table 1:** Specific primers used for RT-PCR

Gene	Forward	Reverse
GAPDH	5′-ATGGGGAAGGTGAAGGTCG-3′	5′-GGGGTCATTGATGGCAACAATA-3′
Col I	5′-CCGCCGCTTCACCTACAGC-3′	5′-TTTTGTATTCAATCACTGTCTT-3′
OCN	5′-AGCAAAGGTGCAGCCTTTGT-3′	5′-GCGCCTGGGTCTCTTCACT-3′
ALP	5′-GTTCAGCTCGTACTGCATGTC-3′	5′-ATCGCCTACCAGCTCATGCAT-3′
RunX-2	5′-ATTCCTGTAGATCCGAGCACC-3′	5′-GCTCACGTCGCTCATTTTGC-3′

*GAPDH* glyceraldehyde-3-phosphate dehydrogenase, *Col I* collagen type I, *OCN* osteocalcin, *ALP* alkaline phosphate, *RunX-2* runt-related transcription factor 2

### Statistics

All data are reported as mean ± standard deviation (SD). A one-way ANOVA test was used among multiple groups, while Tukey’s post hoc test was used to determine the difference between any two groups using the SPSS software (SPSS Inc., Chicago, IL, USA). A *p* value < 0.05 was considered statistically significant.

## Results

### Preparation of ASC

**Fig. 1:**
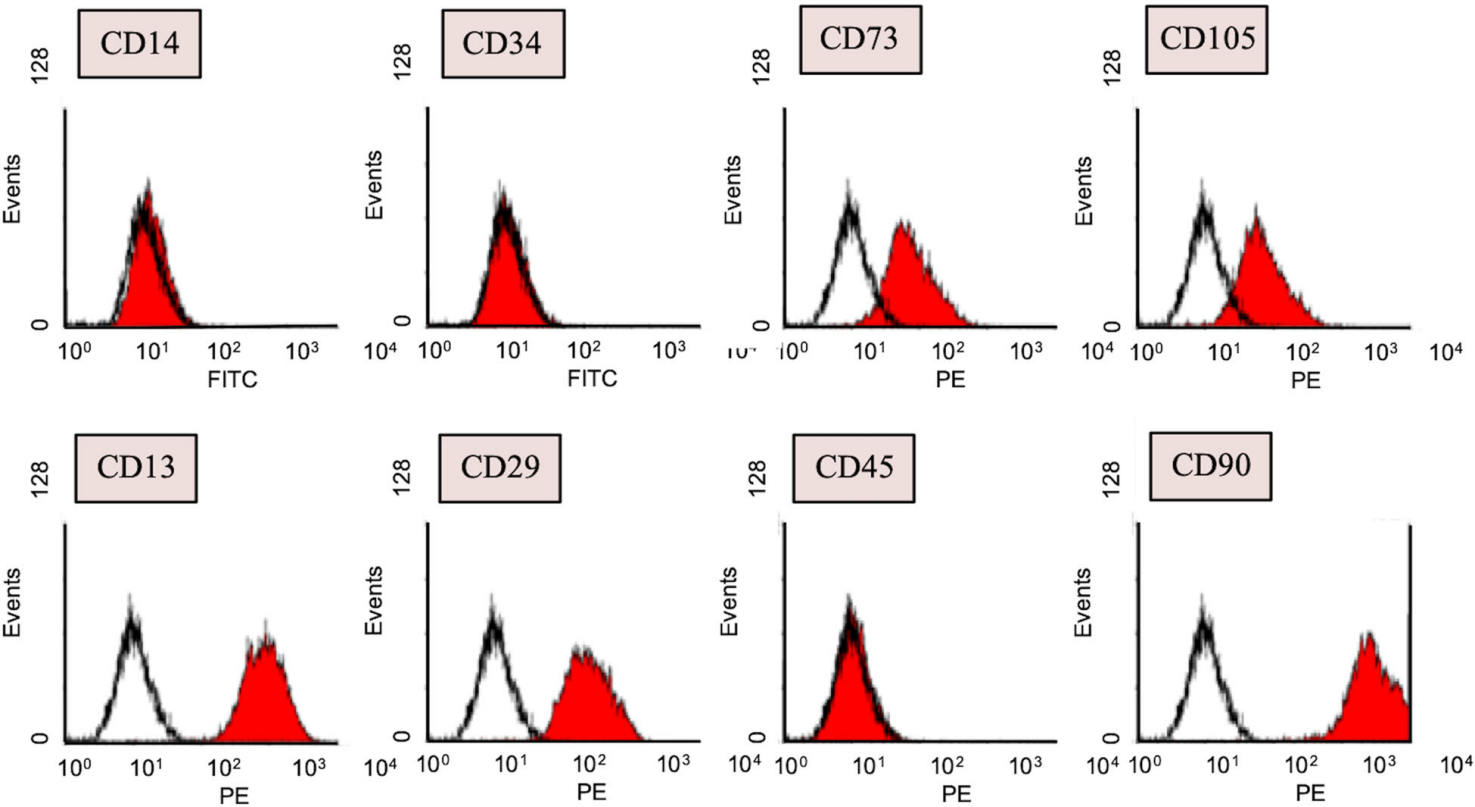
Identification of surface markers on human adipose-derived stem cells (hASCs). The surface markers including CD14, CD34, CD73, CD105, CD13, CD29, CD45, and CD90 were analyzed by flow cytometry, and the numbers were indicated as G-mean values. Each experiment was performed at least three times

**Fig. 2:**
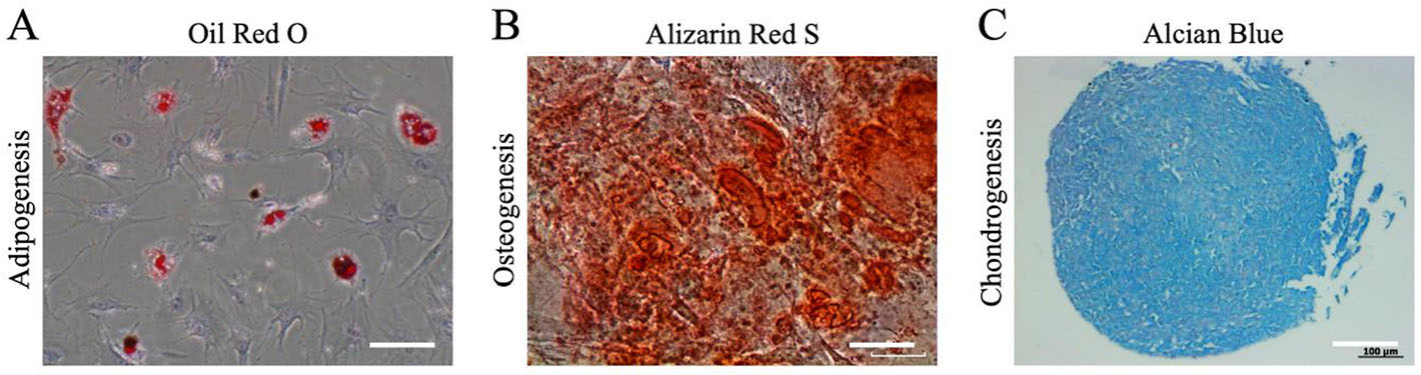
Multilineage differentiation of human ASCs. **a** Adipogenesis was detected using Oil Red O staining, **b** osteogenesis was detected using Alizarin red S staining, and **c** chondrogenesis was detected using Alcian blue. Scale bars = 100 μm

The colonization of spindle-shaped cells from ASCs begins in day 5. Cells were examined at passage 3 by flow cytometry and showed negative of CD14, CD34, and CD 45. Markers of CD13, CD29, CD73, CD90, and CD105 were 100%, 99.89%, 92.64%, 100%, and 90.15% positive, which corresponded to the definition of mesenchymal stem cells according to ISCT (Fig. 1). The isolated cells from those isolated from the fat tissue aspirates could be differentiated to form adipocytes, chondrocytes, and osteocytes when the appropriate growth factors were added (Fig. 2).

### Cell preparation of ASC-iPSCs

**Fig. 3:**
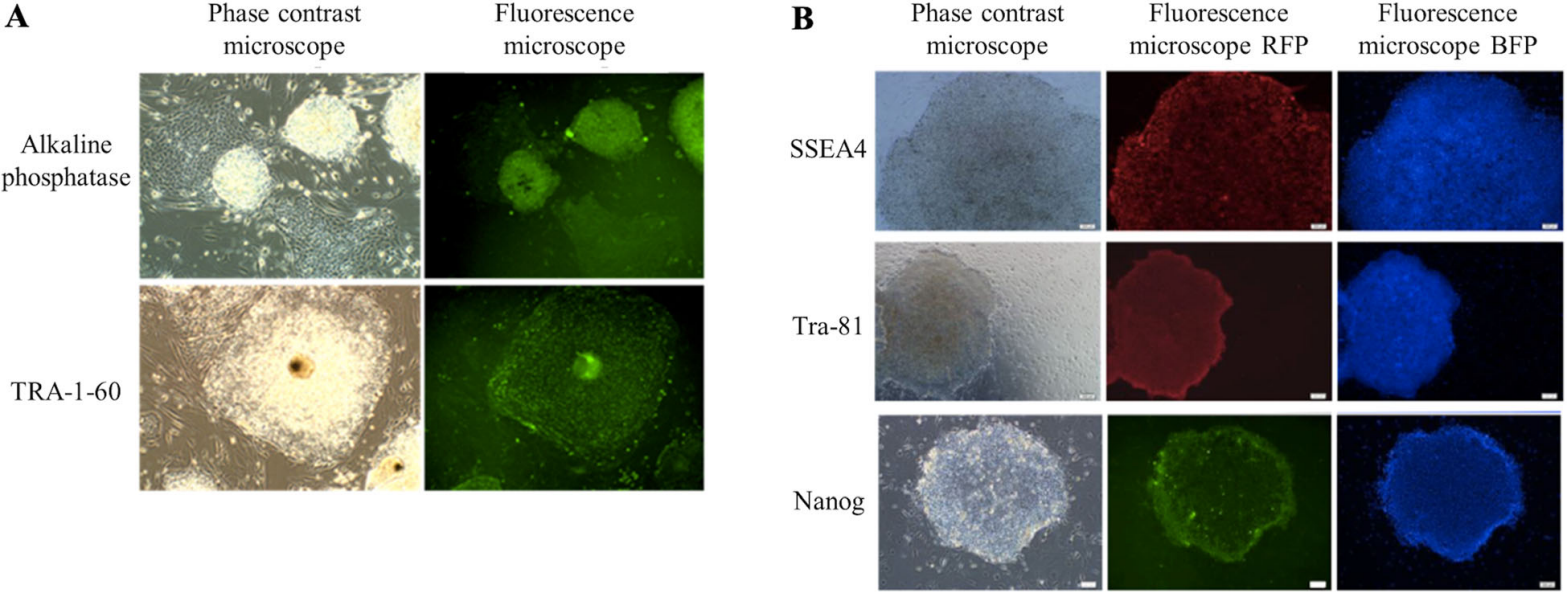
**a** The morphology and embryoid body of iPSCs under a phase contrast and fluorescence microscope. **b** Expression of SSEA-4, Tra-81, and Nanog protein demonstrate the successful induction of iPSCs from ASCs

**Fig. 4:**
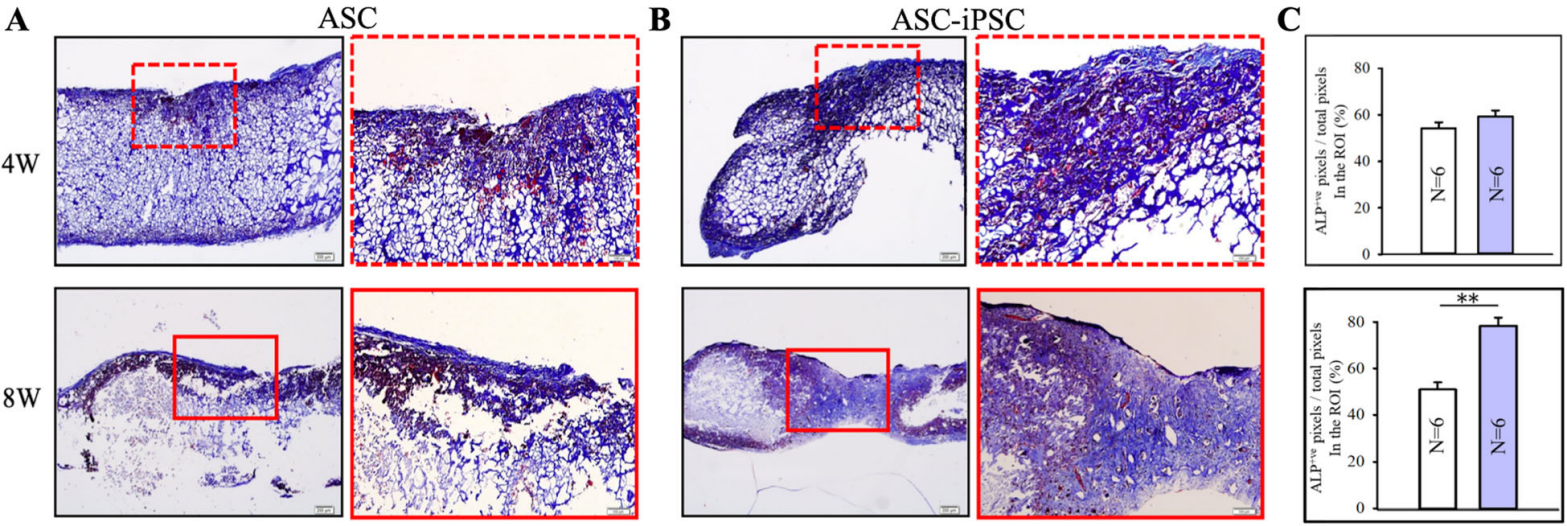
**a** RT-PCR analysis to identify the stemness gene from iPSCs, including Oct-4, Sox2, c-Myc, Klf4, and Nanog. 1 = experimental group; 2 = control group. **b** Identification of teratoma with H&E staining. The structures of the ectoderm, mesoderm, and endoderm were present in the teratoma

The third passage of ASCs was utilized to reprogram to ASC-iPSCs. SSEA-4, Tra-81, and Nanog proteins were all expressed in ASC-iPSCs by immunofluorescence, indicating the embryonic state of the cells (Fig. 3). RT-PCR showed expression of OCT-4 SOX-2, c-Myc, KLF4, and Nanog, whereas the control was all negative (Fig. 4a). Teratoma formation was observed at week 14 of implantation. The histology of specimen revealed formation of ectoderm, mesoderm, and endoderm (Fig. 4b). These evidences suggested the capability of retroviral transfection from ASC to ASC-iPSCs.

### In vivo differentiation

Before in vivo implantation, 1 × 10^6^ cells were seeded onto each cryogel scaffold for 3 days followed by osteo-induction medium for 14 days. At weeks 4 and 8, the scaffolds were retrieved for gross examination, histology, and RT-PCR. One of the crucial characteristics of scaffold is timely degradation to be replaced by migrated cells and surrounding apparatus. From gross examination, the cryogel scaffold provides good stability and degradation with time in vivo.

#### Histology

**Fig. 5:**
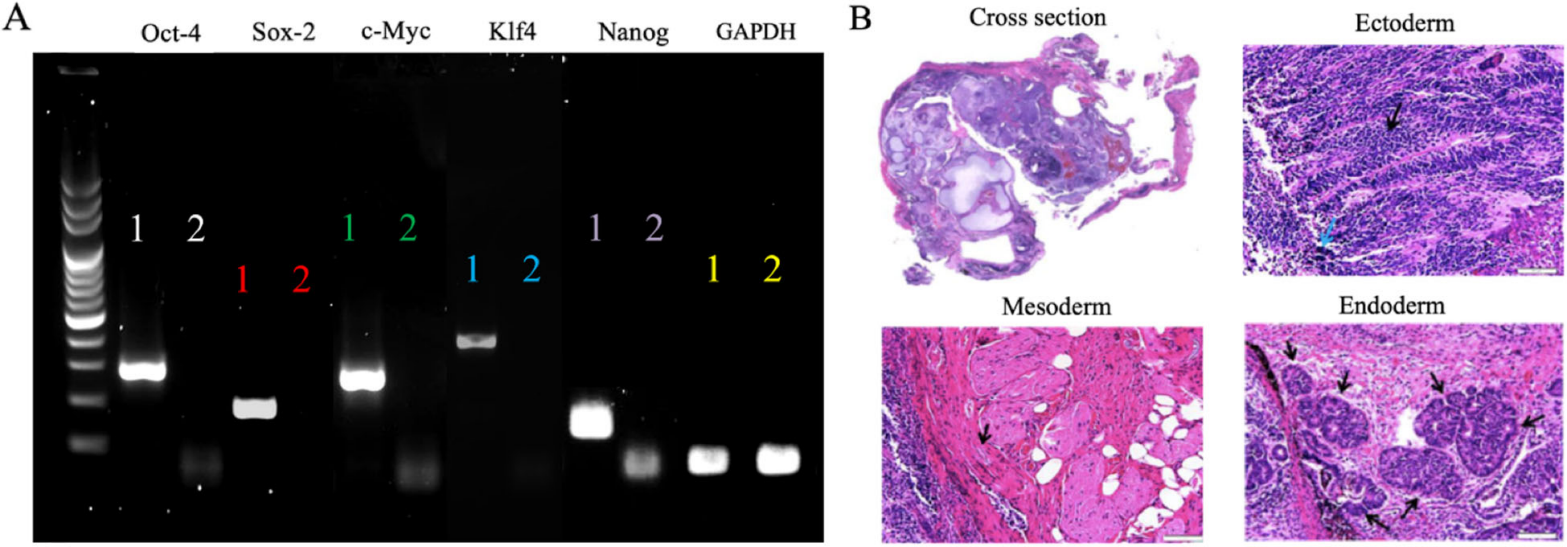
ALP-stained sections 4- and 8-week post-implantation showed lesser osteogenic differentiation in **a** the ASC group than in **b** the ASC-iPSC group. In the ASC-iPSC group, more ALP activity was found at 8 weeks in the central area compared with control ASC groups. **c** Quantification of ALP-positive pixels within the region of interest (**p < 0.01)

The histological evaluation validates the number of osteoblasts in the ASC-iPSC group was higher compared with that in the ASC group in week 8. ASCs showed osteogenic differentiation in week 4 but with limited osteoid secretion, cell expansion, and osteogenic differentiation in week 8, which corresponded to the following results in genetic expression. As for ASC-iPSCs, the level of osteogenic differentiation was similar with ASCs in week 4. In week 8, the ASC-iPSCs differentiate into bone tissue with the existence of osteoids with osteoblasts in the bone matrix, conforming to the osteogenic differentiation in cryogel scaffold (Fig. 5).

#### RT-PCR

**Fig. 6:**
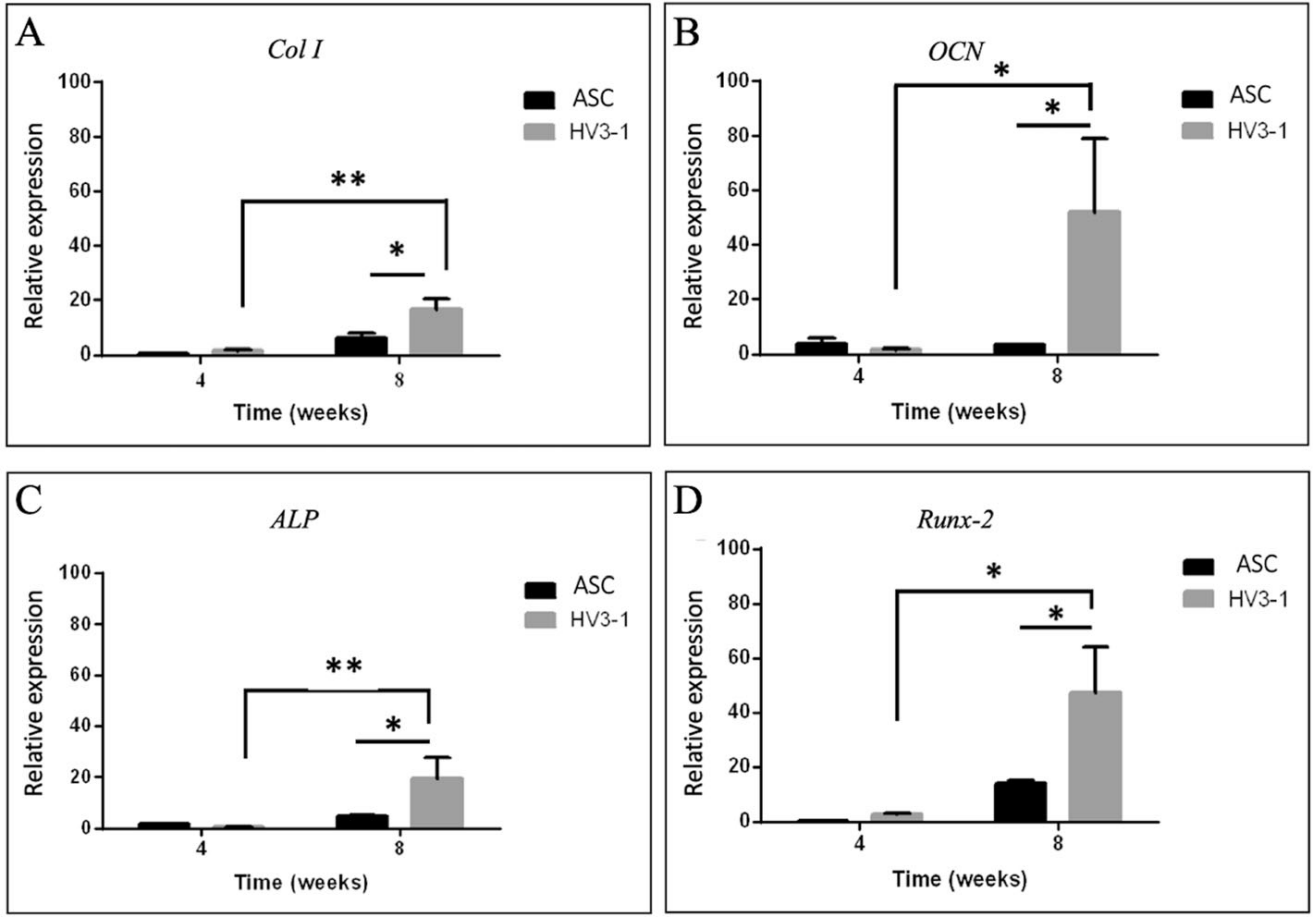
Osteogenic marker gene expression of **a** *Col I*, **b** *OCN*, **c** *ALP*, and **d** *RunX-2* were measured by qRT-PCR and compared between ASC-iPSC and control ASC groups. Relative expressions were normalized to *RPLP1* and *YWHAZ* (housekeeping genes). In the experimental group, expression of genes *Col I*, OCN, *ALP*, and *RunX-2* was significantly increased in comparison to controls at week 8. Data are presented as mean ± SD (*n* = 3), (*p < 0.05; **p < 0.01)

Four genes (Col I, OCN, ALP, and RunX-2) were utilized as markers for bone differentiation via RT-PCR. Expression of Col I and RunX-2 in ASC-iPSCs was significantly higher than that in the ASC group in week 8 (*p* < 0.05). Expression of Col I, ALP, and RunX-2 in ASC-iPSCs were statistically significant from weeks 4 to 8, whereas bone differentiation in ASCs was insignificant (Fig. 6). A trend of superior bone differentiation in the ASC-iPSC group was observed and provides an evidence for greater efficiency of osteogenic differentiation.

## Discussion

Cell origin and quantity are critical in cell therapy and regenerative medicine. ASCs take great advantages of high accessibility, less donor site complications, and abundance in cell numbers. With its potential of osteogenic differentiation, ASCs have been utilized in BTE [4, 5, 17]. However, the efficacy of osteogenic differentiation in ASCs is still inferior to that in BM-MSCs [8]. The purpose of this study is to determine the improvement of osteogenic differentiation from ASC to ASC-iPSCs on cryogel scaffolds. By transcripting Yamanaka factors to ASC, the osteogenic differentiation is enhanced significantly demonstrated by histology and biochemical analysis. The ASC-iPSC yields better osteogenic differentiation compared to ASC in vivo. This is the first report of BTE by utilizing human ASC-iPSCs as a useful cell source for potential clinical applications.

Although ASC has been known with its multilineage differentiation [7], ASC-iPSC demonstrated superior osteogenic differentiation on cryogel scaffolds with significant expression of osteogenetic markers. Three stages were involved in osteogenesis: cell proliferation, extracellular matrix maturation, and mineralization. Col I and ALP were regarded as markers of early differentiation stage whereas OCN is expressed at mid-later stages of osteogenic differentiation. RunX-2 is a transcription factor which acted as an early marker of osteoblastic differentiation and bone formation and as the promoter of several osteogenic genes including collagen type I and OCN. Collagen type I comprises the majority of extracellular matrix in bone formation. At week 8, both RunX-2 and collagen type I in ASC-iPSCs expressed significantly higher levels compared with ASCs, suggesting superior osteogenic differentiation and maturation. OCN plays a crucial role in bone formation and is exclusively produced by osteoblasts, implicating the hemostasis between bone mineralization and calcium ion. Zuk et al. found OCN is the bone-specific gene expressed in osteo-induced ASCs exclusively but not in the non-induced ASCs [7]. Despite not reaching statistical significance, the level of OCN expression in ASC-iPSCs is considered greater in trend compared to ASCs. These results demonstrated that ASC-iPSC possesses superior ability of osteogenic differentiation and osteogenesis regardless of time frame of bone formation.

Little has been known of transcription genes contributing in osteogenic differentiation. We used Yamanaka factors, ectopic expression of OCT4, SOX2, KLF4, and c-MYC, to increase stemness and expansion in ASCs and yield a superior ability of osteogenic differentiation in ASC-iPSCs demonstrated by phenotype and gene upregulation. Hung et al. demonstrated that expression of stemness genes OCT4, Nanog, SAL4, and KLF4 were increased in bone marrow mesenchymal stem cells under hypoxemic status [18]. These changes resulted in superior adipogenesis and osteogenesis. Increased level of OCT4, Nanog, and KLF4 brings the cell status close to embryonic stem cells or iPSCs. Han et al. demonstrated an increase in expansion and stemness in OCT4/SOX2-overexpressed ASCs by liposomal transfection. The OCT4/SOX2-overexpressed ASCs promote the ability of adipogenic and osteogenic differentiation which is demonstrated by phenotypic analyses and upregulation in collagen type I and osteocalcin [19]. These studies suggested that MSCs with overexpression of OCT, SOX2, and KLF4 would increase stemness and enhance the ability of lineage differentiation following appropriate induction. c-MYC is considered as an oncogene and induces tumorigenecity. Nevertheless, the transforming efficiency of iPSCs would decline if c-MYC is not transduced [20, 21].

Low reprogramming efficiency of iPSCs, under 0.01%, was one of the barriers to extensive applications [9, 21, 22]. Moreover, skin fibroblasts require 4 weeks to expand before reprogramming. To overcome this obstacle, cell sources and quantity will be an essential factor. ASCs can be harvested in large quantities of cells with a relatively safe procedure, less invasiveness, and donor site morbidity compared with other stem or progenitor cells. With distinctive epigenetic landscape, ASC preserves the plasticity to differentiate to multilineage cell lines and closer to pluripotent cells compared with terminally differentiated fibroblasts, which is the original cell lines used in reprogramming to iPSCs [10]. These advantages suggest ASCs might be a better candidate for reprogramming in the clinical setting with more efficient and faster regeneration. iPSCs generated from mesenchymal stem cells or progenitor had shown better reprogramming efficiency and equivalent gene profiles to embryonic stem cells compared to fibroblasts [13, 23]. ASCs demonstrated 8- and 38-fold superior reprogramming efficiency than neural stems cells and MEFs [24]. Sun et al. reported that the reprogramming of iPSCs by human ASCs is 20 times more efficient than IMR90 fibroblasts with reprogramming efficiencies of 0.01% on feeder-free Matrigel substrates to 0.2% on feeders with MEFs [10].

From the protocol for iPSC induction by Yamanaka and following reports, co-culture with MEFs is an essential step that maintains proliferation and self-renewal under a pluripotent state [9]. Co-culture raises the concern of contamination of animal cells and serum which leads to non-human surface antigen and limits clinical accessibility. ASCs possess different genetic and epigenetic landscapes and are regarded as an ideal cell source for reprogramming. Sun and Sugii et al. demonstrate the transformation of ASCs to ASC-iPSCs with and without feeder layers. Without an additional process in co-culture, the variability of ASC-iPSCs could also be reduced.

An ideal scaffold for tissue engineering comprises a balanced combination of biodegradability, physical properties, biocompatibility, and bioactivity. There are three main categories of material combinations: polymeric–organic, ceramic–inorganic, and inorganic–organic composites. Nature bone extracellular matrix consists of inorganic and organic components, in which nHAP takes as a majority in the inorganic portion. nHAP has been integrated for BTE because of its superior bioactivity, biocompatibility, osteoconductivity, osteointegration, and non-toxicity [16, 25]. Rodrigues et al. suggested an osteo-conduction effect of nHAP by demonstrating the increase in cell proliferation of osteoblast-like cells MG63 in collagen-nHAP cryogel scaffold compared to collagen alone [25]. Gelatin is a water-soluble multipeptide polymer that is obtained by hydrolyzing collagen from animal skin and tissues. The arginine-glycine-aspartic acid (RGD) sequence of gelatin allows for better cell attachment and bioactivity [5, 17, 26, 27]. Moreover, different materials and growth factors could be fabricated additionally into cryogel scaffolds to alter physical characteristics and cellular stimulation. In this study, we applied the nHAP-gelatin cryogel which behaves like a sponge and is soft but with strong mechanical characteristics and excellent biocompatibility. The cryogel also possesses the advantage of plasticity and tailoring especially in areas like craniofacial regions which required dedicated manipulations. Previous studies demonstrated a great potential of cryogel scaffolds in tissue engineering of the bone [5, 16, 17, 25, 28], adipose tissue [6], and cartilage [26].

Tumorigenicity is a major concern and limitation in cell-based transplantation and tissue engineering. The differentiated cells following specific induction eliminate the pluripotency and were guided into a less proliferative state which reduces the risk of tumorigenicity. In clinical utilization, genome integrity and in vivo tumorigenicity tests are considered to be essential before implantation, especially for highly proliferative graft cells [29]. Expression of c-MYC might increase the concern of tumorigenicity hindering clinical applications [30]. Nakagawa et al. generated iPSCs without c-MYC. However, the efficiency would decrease to 0.02–0.0002% if c-MYC gene is not transduced [20, 21]. Elimination of c-MYC gene could warrant for further notice and examine the efficacy if certain lineage of cell differentiation is designated.

## Conclusion

ASC is an essential cell source for BTE with benefits of high volume and less-invasive acquisition. With additional transforming Yamanaka factors, ASC-iPSCs showed higher osteogenic differentiation by phenotypic results and elevated expression of Col I and RunX-2. This report suggests that ASC-iPSCs could be an alternative cell source in BTE with better differentiation efficacy for future clinical applications.

## Data Availability

The datasets generated during and/or analyzed during the current study are included within the article and are available from the corresponding authors on reasonable request.

## Abbreviations

iPSCs: Induced pluripotent stem cells
ASCs: Adipose-derived stem cells
BTE: Bone tissue engineering
Col I: Collagen type I
OCN: Osteocalcin
ALP: Alkaline phosphate
RunX-2: Runt-related transcription factor 2
BM-MSCs: Bone marrow mesenchymal stem cells
MEFs: Mouse embryonic fibroblasts
ISCT: International Society for Cellular Therapy
DMEM: Dulbecco’s modified Eagle’s medium
KSR: Knockout serum replacement
H&E: Hematoxylin and eosin
nHAP: Nanohydroxyapatite
